# Congenital Methemoglobinemia in a 33-Year-Old Patient: A Case Report on a Rare Presentation and a Review of the Literature

**DOI:** 10.7759/cureus.35974

**Published:** 2023-03-10

**Authors:** Maya Aldeeb, Ibrahim A Khalil, Mohamed A Yassin

**Affiliations:** 1 Department of Medical Education, Family Medicine Residency Program, Hamad Medical Corporation, Doha, QAT; 2 Department of Urology, Hamad Medical Corporation, Doha, QAT; 3 Department of Hematology and Medical Oncology, National Center for Cancer Care and Research, Hamad Medical Corporation, Doha, QAT

**Keywords:** late diagnosis, asian, cyanosis, congenital, methemoglobinemia

## Abstract

Cyanosis and dyspnea are common complaints in adults and have broad differential diagnoses, of which rare ones such as congenital methemoglobinemia should always be considered in the differential diagnosis. Methemoglobinemia might be acquired or congenital. Patients' symptoms vary from severe shortness of breath, mental status changes, and cyanosis to none. The diagnosis of congenital methemoglobinemia is challenging and requires high index of suspension, especially in older patients. In addition, when diagnosed the treatment of congenital methemoglobinemia is oral ascorbic acid which is readily available. We present a rare case of a 33-year-old lady, who had a long history of recurrent episodes of cyanosis, headache, and fatigue. After excluding cardiopulmonary causes, methemoglobin levels were measured and found to be high, and the chart review revealed high levels of methemoglobin in all her previous episodes, without exposure to any offending agent. This raised the concern of a late diagnosis of congenital methemoglobinemia. The patient was treated with a high dose of ascorbic acid resulting in resolution of the symptoms.

Congenital methemoglobinemia is a rare diagnosis that needs a high index of suspicion, especially in adults. A thorough history, physical examination, and multiple laboratory tests are needed to confirm the diagnosis and rule out other causes.

## Introduction

Methemoglobinemia, a form of hemoglobinopathies, is defined as an increased methemoglobin level, where the ferric form of iron is attached to heme instead of ferrous which results in reduction in tissue oxygenation [1]. A percentage of hemoglobin will be oxidized to methemoglobin under any oxidative stress, but this is regulated by special enzymes to keep it in normal individuals at less than 1.5%. Usually, cyanosis appears when methemoglobin levels exceed 1.5 g/dL, around 15% of total hemoglobin; most adults with methemoglobinemia have the acquired type due to exposure to an offending agent. On the other hand, having unexplained methemoglobinemia in an adult should raise the possibility of the congenital type, which is extremely rare.

Three genetic causes lie behind congenital methemoglobinemia - an autosomal recessive CYB5R3 gene pathogenic variations causing cytochrome b5 reductase deficiency, an autosomal dominant point mutation in alpha-globin gene causing hemoglobin M, or an autosomal recessive NADH-cytochrome b5 reductase deficiency which is extremely rare [2]. We present a rare case of type 1 congenital methemoglobinemia in a 33-year-old female with recurrent episodes of cyanosis treated successfully with ascorbic acid.

This article was previously presented as a poster at the 12th Eurasian Hematology-Oncology Congress on November 10, 2021.

## Case presentation

A 33-year-old lady of Indian origin presented to the emergency department with difficulty in breathing and bluish discoloration of her fingers and lips gradually increased over a period of three days. There was no associated fever, wheezing, or cough. Physical examination showed a conscious alert lady with central and peripheral cyanosis. A focused chest examination revealed normal breathing and heart sounds. Her vital signs at presentation to the emergency department were as follows: SpO_2_ was 90% on room air, heart rate (HR) was 77 beats/minute, blood pressure (BP) was 105/80 mmHg, and respiratory rate (RR) was 20 breaths/min.

Patient’s past medical and surgical history is unremarkable apart from similar but milder episodes of cyanosis with high levels of methemoglobin. The patient is a housewife and non-smoker. Her family history is positive for similar attacks of episodes of cyanosis in her sister.

**Figure 1:**
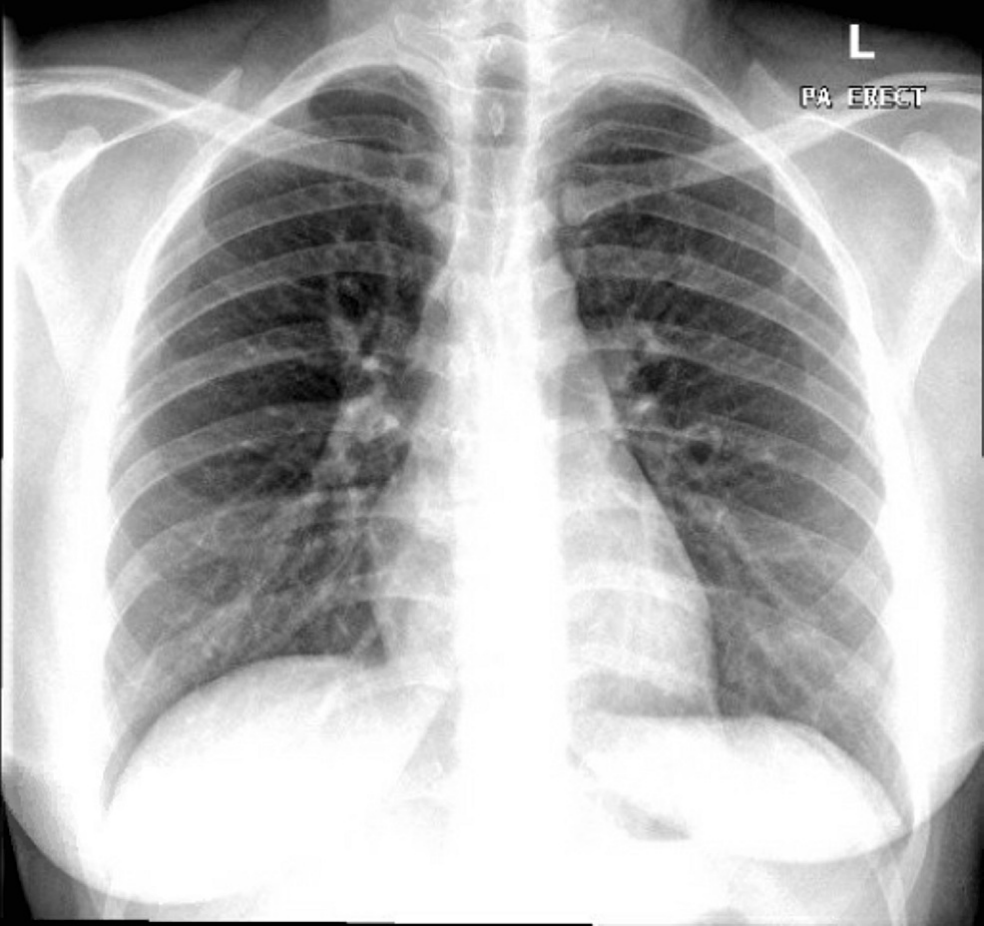
Chest x-ray at presentation.

Blood tests at presentation showed a methemoglobin level of 20.9% (normal range: 0-1.5%). Hemoglobin electrophoresis and peripheral blood smear did not detect any abnormalities. Also, other laboratory tests including complete blood count, glucose-6-phosphate dehydrogenase (G6PD) scan, and arterial blood gases (ABG) results were within normal ranges as shown in Table 1. Electrocardiography showed normal sinus rhythm. Chest radiography showed clear lung fields and a heart of normal size and contour as seen in Figure 1.

**Table 1:** Laboratory results at presentation. pH: potential of hydrogen (blood acidity); PaO_2_: partial pressure of oxygen; PCO_2_: partial pressure of oxygen dioxide, HCO_3_: bicarbonate; O_2 _sat: oxygen saturation in blood; WBC: white blood cell count; RBC: red blood cell count; Hgb: hemoglobin; Hct: hematocrit; MCV: mean cell volume; PLT: platelets count

Laboratory test	Result	Reference range
pH	7.41	7.35-7.45
PaO_2_	160 mmHg	83-108 mmHg
PCO_2_	42 mmHg	35-45 mmHg
HCO_3_	26 mmol/L	23-29 mmol/L
O_2 _sat	96%	95-99%
WBC	9.1×10^3^/uL	4-10×10^3^/uL
RBC	5.1×10^6^/uL	3.8-4.8×10^6^/uL
Hgb	15.9 g/dL	12-15 g/dL
Hct	48.9%	36-46%
MCV	95.9 fL	83-101 fL
PLT	280×10^3^/uL	150-400×10^3^/uL

The patient's clinical picture of recurrent cyanosis, high methemoglobin level, discrepancy between partial pressure of oxygen (PaO_2_) and O_2_ saturation on oximeter, absence of cardiopulmonary causes of cyanosis, and her family history of similar events raised the suspicion of delayed diagnosis of methemoglobinemia. Accordingly, the patient was treated with high dose of ascorbic acid resulted in resolution of the symptoms which confirmed the diagnosis. Upon follow-up, the condition was stable and controlled by ascorbic acid.

## Discussion

Methemoglobinemia is a result of defective regulation of the methemoglobin level by the responsible pathways after oxidative stress. In normal individuals, methemoglobin results from oxidation of ferrous iron that binds to heme to ferric iron, which decreases its ability to bind to oxygen, leading to less oxygen delivery to tissues and left shift in oxygen dissociation curve [1]. Affected individuals can have cyanosis, but clinically significant tissue hypoxia is unusual as compensatory erythrocytosis improves oxygen delivery.

Methemoglobinemia might be acquired or congenital. For acquired methemoglobinemia, a myriad of causes has been described in the literature, for instance, medication such as dapsone, lidocaine, nitrates, and sulfa drugs are triggers [3-5]. The clinical consequences depend upon methemoglobin levels in the blood; symptoms can start at a level >10%, nausea and tachycardia occur at level 30-50%, while a 50% level leads to neurological deterioration. Higher levels can cause arrhythmia, while more than 70% of methemoglobin is considered fatal [2,3]. The acquired form's symptoms are affected by the speed of the increase in the levels and the half-life of the causative agent. Mostly those patients require treatment with intravenous methylene blue.

Conversely, congenital methemoglobinemia causes milder presentation as it is a chronic elevation in methemoglobin with a physiologic compensatory erythrocytosis. Nicotinamide adenine dinucleotide hydrogen (NADH) cytochrome b5 reductase deficiency congenital methemoglobinemia is further classified into two subcategories. Type 1 (enzyme deficiency in the erythrocytes) - those patients are usually asymptomatic or will present late with cyanosis, fatigue, and some shortness of breath-treatment for cosmetic reasons mostly [6]. Type 2 (generalize deficiency of cytochrome b5 in all body tissues) is accompanied by neurological disabilities; however, it is not amenable to treatment at this time.

While acquired methemoglobinemia, triggered by oxidative means, is common, congenital causes are uncommon and rarely documented in the literature [6]. A review of the published English literature on the cases of congenital methemoglobin showed that most of the cases have a high level of methemoglobin and were treated successfully with ascorbic acid as indicated in Table 2.

**Table 2:** The literature review of congenital methemoglobinemia cases. NA: not available; HTN: hypertension; PV: polycythemia vera; CKD: chronic kidney disease; AF: atrial fibrillation

Author	Age	Gender	Nationality	Clinical presentation	Comorbidities	Family history	Hemoglobin	Methemoglobin	Type	Management	Follow-up
Soliman et al. 2018 [6]	29 years	Male	India	Cyanosis and fatigue	Diagnosed with PV and started imatinib	None	20 g/dL	38%	1	Ascorbic acid	Improved
Firouzabadi and Mead et al. 2020 [7]	55 years	Male	NA	Cyanosis, flu-like symptoms	CKD, thyroidectomy due to thyroid cancer AF	None	NA	15%	1	Ascorbic acid	Improved
Ji et al. 2021 [8]	61 years	Male	China	Cyanosis erythrocytosis	High altitude	Parents and one brother have cyanosis	22 g/dL	44.2%	1	Methylene blue	Improved
Ara et al. 2019 [9]	18 years	Male	Bangladesh	Cyanosis Low exercise tolerance	None	None	18 g/dL	36%	1	Ascorbic acid	Improved
Kitao et al. 1974 [10]	36 Years	Female	Japanese	Cyanosis, convulsions	None	None	NA	33%	1	Ascorbic acid	Improved
Aslan et al. 2016 [11]	61 years	Female	Turkish	Mild cyanosis	None	Daughter	NA	16%	1	Ascorbic acid	Improved
Aslan et al. 2016 [11]	17 years	Female	Turkish	Mild cyanosis	None	Mother	NA	12.7%	1	Ascorbic acid	Improved
Londhey et al. 2014 [12]	18 years	Male	India	Cyanosis, headache, bilateral ptosis	None	Sister	NA	37%	1	Ascorbic acid, aspirin	Improved
Tasci et al. 2012 [13]	20 years	Male	NA	Chest pain, exertional dyspnea, cyanosis	None treated as asthma	Brother	NA	40%	1	Methylene blue	Level improved to 8%, cyanosis and other symptoms improved
Hamirani et al. 2008 [14]	21 years	Male	India	Mild cyanosis and exertional dyspnea	None	None	NA	29.5%	1	No treatment	Mild cyanosis
Badawi et al. 2016 [15]	16 years	Male	Arabic	Cyanosis, fatigue, shortness of breath on exertion	None	Brother	17 g/dL	40%	1	Oral vitamin C	Improved
Ramanamurthy 2013 [16]	55 years	Male	India	Cyanosis, weak, syncope	HTN, smoker	None	15 g/dL	30%	1	Vitamin C, riboflavin	Improved
Kedar et al. 2012 [17]	32 years	Female	India	Recurrent early pregnancy loss	None	Positive	14 g/dL	7.5%	1	No indication	NA
Kedar et al. 2012 [17]	34 years	Male	India	None	None	Positive	15 g/dL	2.8%	1	No indication	NA

## Conclusions

Due to mild symptoms, congenital methemoglobinemia is rarely diagnosed and reported as a cause of cyanosis, especially in adults. Despite the benign nature of congenital methemoglobinemia, it is crucial to keep it in the differential diagnosis list when assessing cyanotic patients, mainly if he has a normal PaO_2_. Patients are usually asymptomatic and are treated for cosmetic purposes, but they might suffer from severe complications if exposed to oxidative agents.

In summary, congenital methemoglobinemia is a rare but treatable cause of cyanosis that should be considered in the differential diagnosis of cyanosis.
